# Joint Meeting

**Published:** 1904-09

**Authors:** 


### Society Notes.

JOINT MEETING.—The Milam County Medical Society held a joint meeting with the Bell County Medical Society and the famous Brazos Valley (District) Medical Society at Cameron, August 10th. Dr. J. A. Bodine, of New York, demonstrated some new operations in surgery, especially in varicocele (under local anesthesia). There was a goodly attendance, and much interest. I was not able to be present, but had expected ere now a report of the meeting. Dr. E. N. Shaw, of Cameron, is President, and Dr. I. P. Sessions, of Rockdale, is Secretary.

---

FIFTH DISTRICT MEDICAL SOCIETY, Dr. M. Dugan, President, Dr. M. B. Grace, Secretary, held its second semi-annual meeting at San Antonio, August 25th, with full program and a smoker. No report received.

---

COUNTY MEDICAL SOCIETIES should subscribe for the "Red Back"; they will need it, especially this year. It will keep them informed of all Association matters and of the efforts at medical legislation that the Committee are going to strenously make at the coming session of the Legislature. Every member should be kept posted, so as to co-operate with them. The August number was sent to the secretary of every County Society in Texas; but no more free distribution of the kind will be made. A dollar a year isn't much.

